# Importance of Both Internal and External Iliac Artery Interrogation in Pelvic Trauma as Evidenced by Hemorrhage from Bilateral Corona Mortis with Unilateral Aberrant Origin off the External Iliac Artery

**DOI:** 10.1155/2019/6734816

**Published:** 2019-07-08

**Authors:** Michael Herskowitz, James Walsh, Meghan Lilly, Kimberly McFarland

**Affiliations:** Department of Interventional Radiology, Kings County Hospital, 451 Clarkson Ave, Brooklyn, NY 11203, USA

## Abstract

Transcatheter angiography and embolization has long been recognized as the gold standard for patients with hemodynamic instability secondary to blunt pelvic trauma. While often the bleeding source can be readily localized based on the distribution of extravasation on preprocedural Computed Tomographic Angiography, one should be cautious in assessment for aberrant anatomy. A variant obturator artery originating from the inferior epigastric branch of the external iliac artery is commonly referred to as the corona mortis. We present a case of blunt pelvic trauma in which a patient demonstrated extravasation in the anterior distributions of both internal iliac arteries. Following embolization of bilateral internal iliac arteries, identification and embolization of bilateral corona mortis branches was crucial to achieving hemodynamic stability in this patient.

## 1. Introduction

Patients with hemodynamic instability secondary to hemorrhage in the anterior regions of the pelvis following blunt trauma often stabilize following transcatheter angiography and embolization. The vascular supply in these cases is typically from the internal iliac artery. Despite adequate occlusion of the internal iliac branches, control angiography to evaluate potential contralateral internal iliac, external iliac, and lumbar collateral supply may demonstrate continued hemorrhage. We present an unusual case of embolization of the anterior divisions of both internal iliac arteries as well as bilateral corona mortis artery branches.

## 2. Case Presentation

A 50-year-old male Level I Trauma patient presented following severe blunt trauma in a high-speed motorcycle accident. The patient was initially hemodynamically stable on presentation, with blood pressure of 126/95 and heart rate of 88. He complained of lower abdominal and pelvic pain. Primary survey revealed an unstable pelvis, for which a pelvic binder was placed.

**Figure 1:**
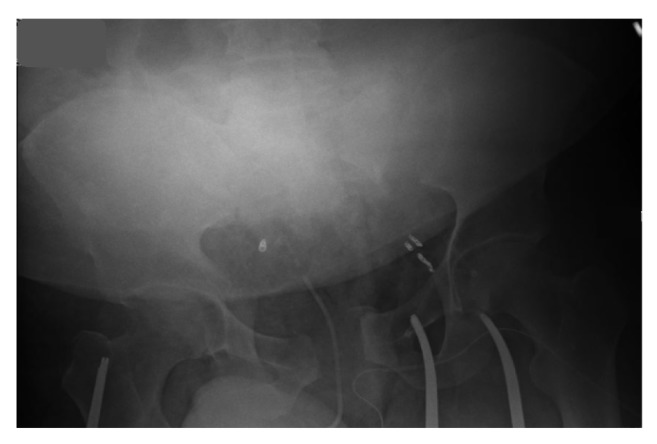
Portable radiograph of the pelvis. There is severe diastasis of the pubic symphysis, avulsion of the left superior pubic ramus, and separation of the left sacroiliac joint.

Preliminary radiographic examinations showed severe diastasis of the symphysis pubis, avulsion fracture of the medial aspect of the left superior pubic ramus, and separation of the left sacroiliac joint (Figure 1). A Computed Tomography Angiogram of the abdomen and pelvis revealed areas of extravasation in the anterior distributions of both internal iliac arteries as well as the posterior division of the left internal iliac artery (not shown).

The patient became hemodynamically unstable while in the Radiology Department, with a blood pressure 91/58 and heart rate 92. Patient's hemoglobin/hematocrit on arrival was 13.2/31.3, though repeat labs revealed a drop to 7.3/22. Transfusion was initiated and the patient was transferred to Interventional Radiology for emergent angiography.

**Figure 2:**
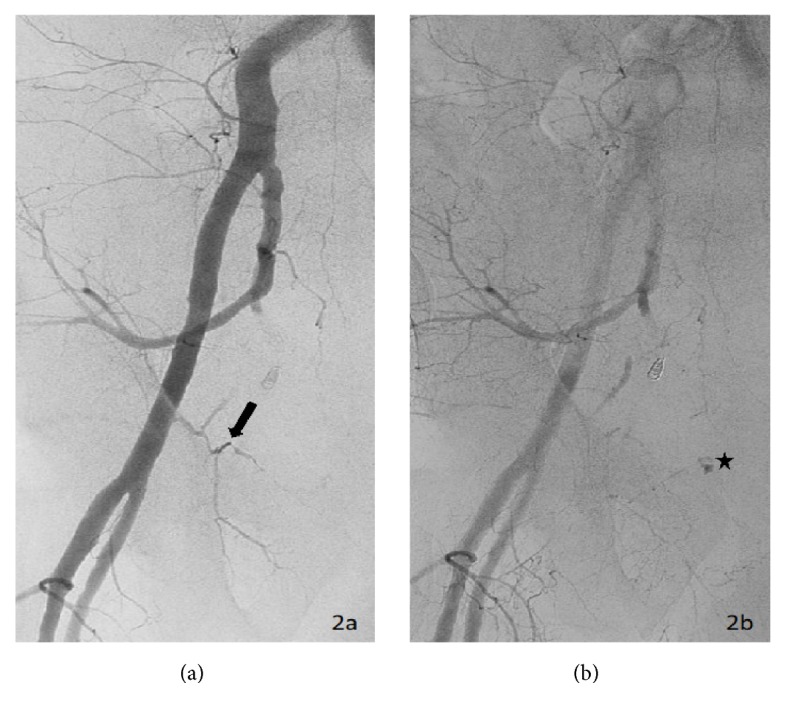
Right external iliac arteriogram. (a) The early phase demonstrates a right corona mortis (arrow) originating from the inferior epigastric artery. (b) Delayed imaging reveals active extravasation from the corona mortis (star).

**Figure 3:**
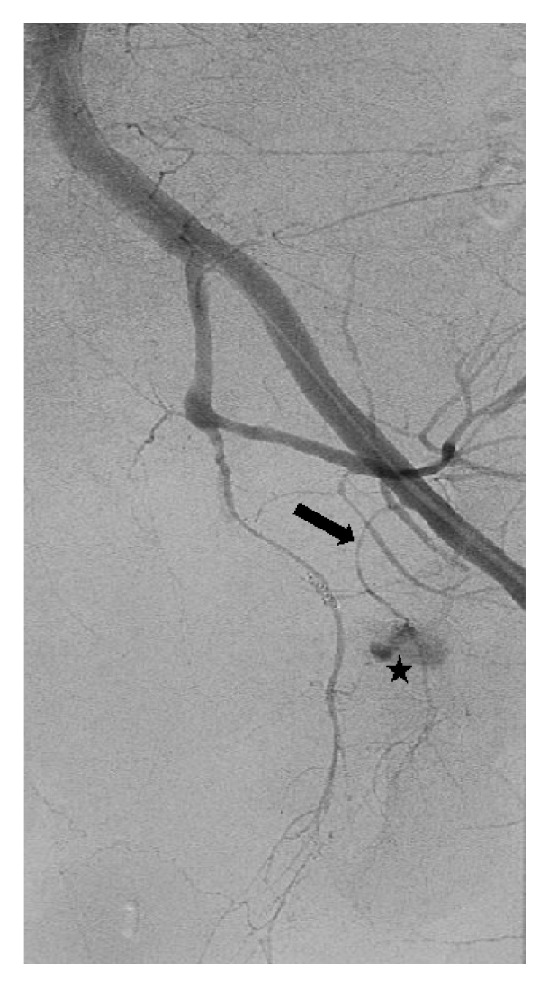
Left common iliac angiogram. Active extravasation (star) is seen from a left corona mortis (arrow). In this case, the internal and external iliac communication arises from an aberrant pubic branch. This aberrant pubic branch originates from the left external iliac artery proximal to the origin of inferior epigastric artery.

**Figure 4:**
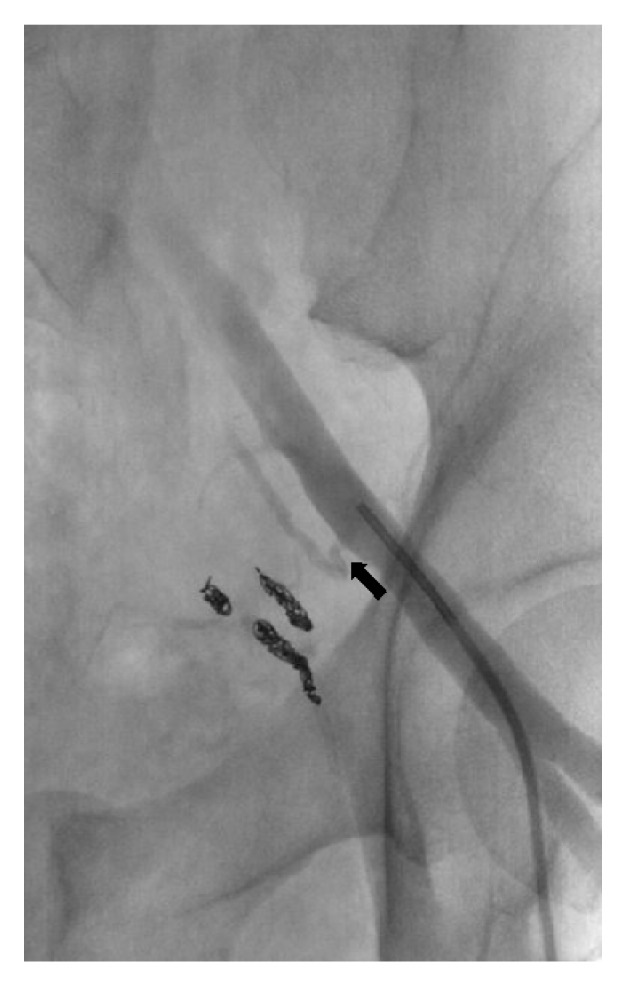
Left common iliac injection. The inferior epigastric artery is clearly seen (arrow). Note absence of a corona mortis or pubic branches originating from the left inferior epigastric artery.

Pelvic aortogram demonstrated areas of contrast extravasation in bilateral inferior pelvic regions (not shown). Bilateral selective internal iliac arteriograms were performed, revealing active extravasation from the anterior divisions on both sides (not shown). Both right and left anterior divisions of the internal iliac arteries were embolized to stasis with gelfoam slurry (not shown). A right external iliac arteriogram showed active extravasation off the corona mortis branch of the right inferior epigastric artery (Figures 2(a) and 2(b)). This branch was embolized to stasis with several 0.018 coils. At this point, the patient remained hypotensive, with a blood pressure of 90/50s. A left external iliac arteriogram was performed, revealing active extravasation from an aberrant pubic branch off the left external iliac artery proximal to the take-off of the left inferior epigastric artery (Figure 3). We proceeded to embolization with 0.018 coils and gelfoam slurry. Once stasis was achieved, the patient became hemodynamically stable, with blood pressure improving to 145/80. No further transfusions were necessary. A selective left inferior epigastric arteriogram confirmed the absence of corona mortis or pubic branches (Figure 4).

Once this was performed, his blood pressure normalized and a completion pelvic angiography revealed resolution of the multiple sites of contrast extravasation. In total, the patient received 6 units of packed red blood cells, 5 packs of fresh frozen plasma, and 1 pack of platelets from arrival to the completion of the procedure.

The patient was transported to the Surgical Intensive Care Unit for further resuscitation. He did not require additional transfusions. The following day he underwent pelvic fixation by orthopedic surgery. He continued to improve clinically following the treatment of his acute injuries and was transferred to a rehabilitation facility for continued care.

## 3. Discussion

Transcatheter embolization is an accepted treatment modality for patients with hemodynamic instability after blunt pelvic trauma [1]. Computed Tomographic Angiography is also valuable, both as a screening tool and as a guide to localize expected sites of involvement during angiography. Significant areas of hemorrhage in the anterior portions of the pelvis are usually supplied by branches of the anterior division of the internal iliac artery. However, Interventional Radiologists must be aware of anatomic variants supplying the retropubic regions of the anterior pelvis. Controversy exists concerning the nomenclature of vascular supply in the retropubic region [2, 3]. The obturator artery is a terminal branch of the internal iliac artery, which supplies branches to that area. 17% of patients have an arterial corona mortis, defined as an anastomotic communication between the external iliac and obturator systems [4]. The external iliac supply may arise from the inferior epigastric, or, rarely, the femoral artery. Whether the anomaly is an obturator artery originating from the external iliac alone (up to 20% incidence [2]) or from both internal and external iliac arteries with a corona mortis anastomosis, transcatheter embolization of hemorrhage in the regions of the pubic bones should include study of bilateral internal and external iliac systems to ensure adequate evaluation of potential collateral supply. This branch may anastomose with the obturator artery originating from the internal iliac artery or may exist in the absence of said branch off the internal iliac artery. This variant obturator artery branch from the external iliac artery is referred to as the “corona mortis.”

We present an unusual case of severe hemodynamic instability after blunt pelvic trauma requiring embolization of both anterior distributions of the internal iliac arteries as well as embolization of bilateral corona mortis arteries off the external iliac arteries. Many authors have reported cases of unilateral corona mortis artery embolization associated with blunt pelvic trauma [5–8]. We believe this is the only reported case of bilateral corona mortis embolization. Interventional Radiologists must be cognizant of the supply of the external iliac artery branches of the retropubic regions.
